# MicroRNA-375/SEC23A as biomarkers of the *in vitro* efficacy of vandetanib

**DOI:** 10.18632/oncotarget.8458

**Published:** 2016-03-29

**Authors:** Sandra Lassalle, Joséphine Zangari, Alexandra Popa, Marius Ilie, Véronique Hofman, Elodie Long, Martine Patey, Frédérique Tissier, Geneviève Belléannée, Hélène Trouette, Bogdan Catargi, Isabelle Peyrottes, Jean-Louis Sadoul, Olivier Bordone, Christelle Bonnetaud, Catherine Butori, Alexandre Bozec, Nicolas Guevara, José Santini, Imène Sarah Hénaoui, Géraldine Lemaire, Olivier Blanck, Philippe Vielh, Pascal Barbry, Bernard Mari, Patrick Brest, Paul Hofman

**Affiliations:** ^1^ Centre Hospitalier Universitaire de Nice, Laboratory of Clinical and Experimental Pathology, Nice, France; ^2^ Institute of Research on Cancer and Ageing of Nice (IRCAN), INSERM U1081/CNRS UMR7284, Nice, France; ^3^ University of Nice Sophia-Antipolis, Nice, France; ^4^ Centre Hospitalier Universitaire de Nice, Hospital Integrated Biobank (BB 0033-00025), Nice, France; ^5^ Fédération Hospitalo-Universitaire “OncoAge”, University of Nice Sophia Antipolis, Nice, France; ^6^ Institut de Pharmacologie Moléculaire et Cellulaire IPMC, CNRS UMR7275, Sophia-Antipolis, France; ^7^ Hôpital Universitaire de Reims - Hôpital Robert Debré, Department of Pathology, Institut Jean Godinot, Reims, France; ^8^ Assistance Publique - Hôpitaux de Paris (AP-HP), Groupe Hospitalier Pitié-Salpêtrière, Laboratory of Pathology, Paris, France; ^9^ Centre Hospitalier Universitaire de Bordeaux, Hôpital Universitaire de Pessac-Haut Lévêque, Laboratory of Pathology, Pessac, France; ^10^ Centre Hospitalier Universitaire de Bordeaux, Department of Endocrinology, Pessac, France; ^11^ Centre Antoine Lacassagne, Laboratory of Pathology, Nice, France; ^12^ Centre Hospitalier Universitaire de Nice, Hôpital de l'Archet, Department of Endocrinology, Nice, France; ^13^ Centre Antoine Lacassagne, Head and Neck Institute, Surgery and Otorhinolaryngology Department, Nice, France; ^14^ Bayer CropScience SA, Research Center, Sophia Antipolis, Valbonne, France; ^15^ Institut Gustave Roussy, Translational Research Laboratory, Department of Pathology, Villejuif, France

**Keywords:** microRNA, medullary thyroid carcinoma, microRNA-375, treatment, vandetanib

## Abstract

In this study, we performed microRNA (miRNA) expression profiling on a large series of sporadic and hereditary forms of medullary thyroid carcinomas (MTC). More than 60 miRNAs were significantly deregulated in tumor *vs* adjacent non-tumor tissues, partially overlapping with results of previous studies. We focused our attention on the strongest up-regulated miRNA in MTC samples, miR-375, the deregulation of which has been previously observed in a variety of human malignancies including MTC. We identified miR-375 targets by combining gene expression signatures from human MTC (TT) and normal follicular (Nthy-ori 3-1) cell lines transfected with an antagomiR-375 inhibitor or a miR-375 mimic, respectively, and from an *in silico* analysis of thyroid cell lines of Cancer Cell Line Encyclopedia datasets. This approach identified SEC23A as a *bona fide* miR-375 target, which we validated by immunoblotting and immunohistochemistry of non-tumor and pathological thyroid tissue. Furthermore, we observed that miR-375 overexpression was associated with decreased cell proliferation and synergistically increased sensitivity to vandetanib, the clinically relevant treatment of metastatic MTC. We found that miR-375 increased PARP cleavage and decreased AKT phosphorylation, affecting both cell proliferation and viability. We confirmed these results through SEC23A direct silencing in combination with vandetanib, highlighting the importance of SEC23A in the miR-375-associated increased sensitivity to vandetanib.

Since the combination of increased expression of miR-375 and decreased expression of SEC23A point to sensitivity to vandetanib, we question if the expression levels of miR-375 and SEC23A should be evaluated as an indicator of eligibility for treatment of MTC patients with vandetanib.

## INTRODUCTION

Thyroid carcinomas are the most common cancer of the endocrine system [1]. Among these tumors, medullary thyroid carcinoma (MTC) is a rare calcitonin-producing tumor, which arises from thyroid gland parafollicular C cells and accounts for 3-8% of all thyroid carcinomas [2]. Most cases of MTC are sporadic (SMTC), whereas the remaining cases are due to hereditary forms (HMTC), caused by germline activating mutations of the *RET* proto-oncogene [3]. Somatic *RET* gene mutations can also be found in 40-50% of SMTC [4].

MTC are aggressive tumors, for which lymph node metastases are found in 55% of MTC patients at diagnosis [5]. Currently, surgery is the treatment of choice for MTC, consisting in total thyroidectomy and lymph node dissection. However, despite surgery, 50% of patients with MTC relapse [6]. Metastatic and refractory MTC are relatively unresponsive to radiation therapy and to standard chemotherapeutic regimens [7]. Recently, multi-kinase inhibitors have been tested for treatment of advanced MTC [8]. In particular, vandetanib has been recently approved for treatment of patients with recurrent or metastatic unresectable MTC [9, 10].

MicroRNA (miRNA) are small non-coding RNA gene products that have important regulatory functions on basic cellular processes like development, differentiation, proliferation and cell death, affecting major biological domains such as stemness, immunity and cancer [11, 12]. MiRNAs mediate direct post-transcriptional silencing of complementary mRNA targets through association with a large miRNA-induced silencing complex (miRISC). Recent advances have indicated that target silencing is carried out by a combination of translational repression and mRNA destabilization, with the latter contributing to most of the steady-state repression in animal cell cultures. MiRNAs can function as tumor suppressors or oncogenes [13] and alteration in their expression plays a critical role in tumorigenesis, bringing new diagnostic and therapeutic opportunities [12]. MiRNAs of thyroid tumors have been extensively studied by us and others [14–18], for review see Pallante *et al*. [19]. Several studies have reported miRNA profiling in MTC [20–26].

The aim of our study was to identify the miRNA expression profile of a large cohort of paired tumor and normal tissue of MTC patients (62 cases), and to investigate miRNA targets that may be important for cancer theragnosis. Thus we modulated the expression of the most up-regulated miRNA, miR-375, in different tumor cell lines and identified SEC23A as a *bona fide* miR-375 target in MTC through a combination of *in silico* and experimental approaches. Finally, we analyzed the impact of this miR-375/SEC23A axis on cell proliferation and viability especially in association with vandetanib, a clinically relevant cancer drug for treatment of metastatic MTC patients.

## RESULTS

### Specific microRNA expression profiles of MTC at diagnosis

**Figure 1:**
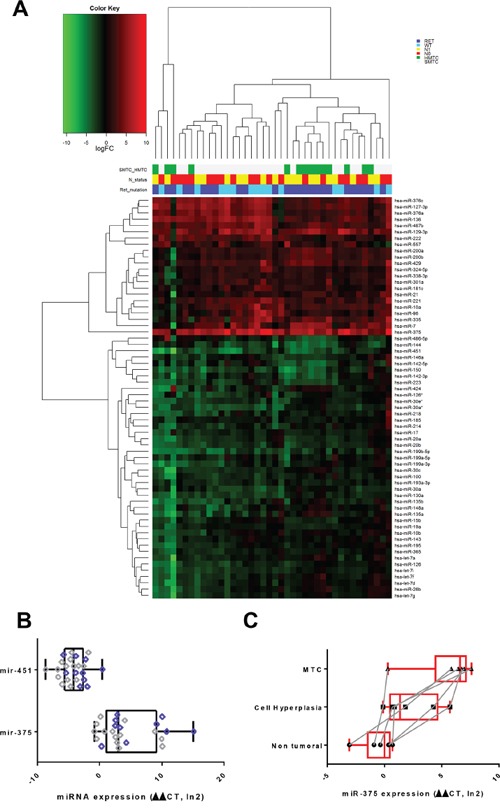
MiRNA expression in patients with MTC **A.** Hierarchical clustering on logFC expression of the 64 differentially expressed miRs. The mutational SMTC and HMTC status, the presence or absence of *RET* gene mutations, as well as the N status are color coded for all patients. The logFC individual gene colors are coded with a gradient from green (under-expression) to red (over-expression). MTC: Medullary Thyroid Carcinoma. HMTC: Hereditary Medullary Thyroid Carcinoma. SMTC: Sporadic Medullary Thyroid Carcinoma. **B.** Semi quantitative real-time PCR validation of the miRNA microarray results. Relative expression of miR-375 and miR-451 in tumor *vs* non-tumor tissue of 22 patients (11 HMTC, 11 SMTC). RNU19 normalized. Patient harbouring RET mutation are indicated in violet dots.*= *P*-value ≤ 0.05. **C.** Semi quantitative real-time PCR validation of the miRNA microarray results. Relative expression of miR-375 in non-tumor adjacent tissue, C-Cell hyperplasia and MTC of 6 patients bearing these pathologies in their thyroid. The dots corresponding to the same patient in the different tissues are connected with a grey line RNU19 was used as reference and values were normalized given the percentage of C-cell quantification in the tissue (hyperplasia, MTC).

The microRNA expression profiles were first determined for 40 MTC, corresponding to 14 HMTC with germinal *RET* mutations, and 26 SMTC with (11 cases) or without (15 cases) somatic *RET* mutations. The main epidemiological, clinical and pathological associated data are summarized in Table 1 and 2. Sixty-four miRNA were significantly modulated in tumor *vs* non-tumor samples (average intensity >6, Log2 Ratio >1 or <-1, adj. P. val<=0.05) (Figure 1A, Table 3). MiR-375 was the most up-regulated and miR-451 the most down-regulated. We selected these 2 miRNAs for further validation by qPCR in 22 MTC (11 HMTC and 11 SMTC). As expected, the validation set yielded over-expression of miR-375 and under-expression of miR-451 in tumor *vs* non-tumor tissues (Figure 1B). We also found that the miR-375 expression gradually increased with disease progression (Figure 1C), even after an adjustment for the percentage weight based on the estimation of the C-cell content by calcitonin and haematoxylin staining.

**Table 1:** Clinical features of the patient training cohort

Patients	Sex	Age at diagnosis	MEN	Germinal *RET* mutation	Somatic 918 *RET* mutation	Surgical treatment	Follow-up (months)	Persistent and/or recurrent disease	Deceased
1	M	48	no	no	no	TT + LND + RT	34	persistent	yes
2	F	43	no	no	no	TT + LND	68	no	no
3	F	39	no	no	no	TT + LND	75	no	no
4	F	36	no	no	yes	TT + LND	71	no	no
5	M	84	no	no	no	TT + LND	78	Persistent and recurrent	no
6	F	57	no	no	yes	TT + LND	70	no	no
7	F	73	no	no	no	TT + LND	69	no	no
8	F	38	no	no	no	TT + LND	60	no	no
9	M	37	no	Exon 15 codon 891	NS	TT + LND	60	no	no
10	M	76	no	no	no	TT + LND	34	Persistent and recurrent	yes
11	F	65	no	Exon 13 codon 790	NS	TT + LND	43	persistent	no
12	F	73	no	no	yes	TT + LND	39	no	no
13	F	49	no	no	no	TT + LND	66	no	no
14	F	65	no	no	no	TT + LND	38	persistent	no
15	F	67	no	no	yes	TT + LND	75	no	no
16	F	45	no	no	no	TT + LND	63	Persistent and recurrent	no
17	F	60	no	no	no	TT + LND	52	no	no
18	F	59	no	no	yes	TT + LND	24	no	no
19	M	61	FMTC	Exon 10 codon 611	NS	TT + LND	1	no	no
20	F	58	MEN 2A	Exon 10 codon 611	NS	TT + LND	24	no	no
21	F	65	no	no	no	TT + LND	21	no	no
22	F	60	no	no	no	TT + LND	238	no	no
23	M	35	MEN 2A	Exon 10 codon 618	NS	TT + LND	234	no	no
24	F	67	no	no	no	TT + LND	45	no	no
25	F	70	no	Exon 14 codon 804	NS	TT + LND	31	persistent	no
26	M	46	no	Exon 10 codon 618	NS	TT + LND	43	no	no
27	M	45	no	no	yes	TT + LND	4	no	no
28	M	49	NA	no	yes	TT + LND	24	recurrent	yes
29	M	38	MEN 2A	Exon 11 codon 634	NS	TT + LND	199	recurrent	no
30	F	52	no	no	yes	TT + LND	74	persistent	no
31	M	72	MEN 2A	Exon 10*	NS	TT + LND	26	persistent	no
32	F	65	MEN 2A	Exon 10*	NS	TT + LND	43	no	no
33	F	50	MEN 2	Yes*	NS	TT + LND	31	no	no
34	F	64	MEN 2	Exon 10 codon 618	NS	TT + LND	60	Persistent	no
35	F	41	MEN 2	Exon 10*	NS	TT + LND	48	Persistent and recurrent	no
36	M	65	no	no	yes	TT + LND	40	persistent	no
37	M	46	MEN 2	Exon 10 codon 618	NS	TT + LND	2	persistent	no
38	F	36	no	no	no	TT + LND	24	no	no
39	M	37	no	no	yes	TT + LND	62	persistent	no
40	F	75	no	no	yes	TT + LND	2	NA	NA

* not otherwise specified; FMTC: Familial Medullary Thyroid Carcinoma; MEN: Multiple Endocrine Neoplasia; NS: Not Searched; NA: Not Available; TT: Total Thyroidectomy; LND: Lymph Node Dissection; RT: Radiotherapy; Persistent disease: high calcitonin 3 months after initial surgery; Recurrent disease: clinical and biochemical cure at 3 months but disease recurrence thereafter.

**Table 2:** Pathological features of the medullary thyroid carcinomas (training set)

Patients	Tumor localization	Tumor size (cm)	Percent of tumor cells	Mitotic activity	Necrosis (%)	Amyloid deposits	Vascular invasion	ETE	Multi-centri-city	CCH	Lymph node metastasis at diagnosis	Lymph node capsular effraction	Distant metastasis at diagnosis	pTNM			Stage
														pT	pN	pM	
1	Thyroid	0.7	40	<2	0	yes	yes	yes	yes	no	yes	yes	no	4a	1b	0	IVA
2	Thyroid	2.5	60	2-10	0	yes	yes	no	no	no	no	NA	no	2	0	0	I
3	Thyroid	0.7	80	<2	0	yes	no	no	no	no	no	NA	no	1	0	0	I
4	thyroid	1.4	60	<2	0	yes	no	no	no	no	no	NA	no	1	0	0	I
5	Thyroid	1.3	25	<2	<10	yes	yes	yes	no	yes	yes	yes	Yes (lung)	4a	1b	1	IVC
6	Thyroid	0.6	40	<2	<10	yes	no	no	no	no	no	NA	no	1	0	0	I
7	Thyroid	0.9	25	<2	0	yes	yes	no	no	no	no	NA	no	1	0	0	I
8	Thyroid	1.5	70	<2	0	no	yes	no	no	no	no	NA	no	1	0	0	I
9	Thyroid	0.4	80	<2	0	yes	no	no	yes	yes	no	NA	no	1	0	0	I
10	Thyroid	2.5	40	2-10	0	yes	yes	yes	no	no	yes	yes	no	3	1b	0	IVA
11	Thyroid	1.7	50	<2	0	yes	yes	yes	yes	yes	yes	yes	no	3	1b	0	IVA
12	Thyroid	1.4	50	<2	0	yes	yes	no	no	no	yes	yes	no	1	1a	0	III
13	Thyroid	0.5	80	<2	0	no	no	no	no	no	no	NA	no	1	0	0	I
14	Thyroid	1.4	70	<2	<10	yes	yes	yes	no	no	yes	yes	no	3	1b	0	IVA
15	Thyroid	3	80	<2	0	yes	no	no	no	yes	no	NA	no	2	0	0	II
16	Thyroid	3	60	<2	0	no	yes	no	yes	no	yes	yes	no	2	1b	0	IVA
17	Thyroid	0.9	80	<2	0	yes	no	no	no	yes	no	NA	no	1	0	0	I
18	Thyroid	1.5	30	<2	0	no	no	no	yes	yes	no	NA	no	1	0	0	I
19	Thyroid	1.2	40	<2	0	yes	no	no	yes	yes	yes	yes	no	1	1b	0	IVA
20	Thyroid	2	80	<2	0	yes	no	no	yes	no	no	no	no	1	0	0	I
21	Thyroid	1.6	80	<2	0	no	no	no	no	no	no	no	no	1	0	0	I
22	Thyroid	2.5	NA	2-10	0	yes	no	no	no	no	no	no	no	2	0	0	II
23	Thyroid	3	NA	<2	0	no	no	no	yes	yes	no	no	no	2	0	0	II
24	Thyroid	1.8	NA	2-10	0	yes	no	no	no	no	no	no	no	1	0	0	I
25	Thyroid	1.9	NA	NS	0	no	no	no	no	yes	yes	no	no	1	1b	0	IVA
26	Thyroid	0.8	NA	0	0	yes	no	no	yes	yes	no	no	no	1	0	0	I
27	Thyroid	1	NA	0	0	no	no	no	no	no	yes	no	no	1	1a	0	III
28	Thyroid	4.5	NA	2-10	<50	no	yes	yes	no	no	yes	yes	no	3	1b	0	IVA
29	Thyroid	NA	NA	NS	no	NA	yes	NA	no	no	yes	NA	NA	NA	1	NA	NA
30	Thyroid	2.2	NA	NS	no	yes	yes	yes	no	no	yes	NA	no	3	1b	0	IVA
31	Thyroid	3	NA	NS	no	yes	no	no	yes	yes	yes	yes	no	1	1a	0	III
32	Thyroid	0.8	NA	NS	no	no	no	no	yes	yes	no	no	no	1	0	0	I
33	Thyroid	0.5	10	<2	no	no	no	no	yes	yes	no	no	no	1	0	0	I
34	Thyroid	3	75	<2	no	no	yes	no	yes	no	yes	yes	no	2	1a	0	III
35	Thyroid	3	30	<2	no	no	yes	no	yes	yes	yes	yes	no	2	1b	0	IVA
36	Thyroid	2.3	75	<2	no	yes	yes	yes	no	yes	no	no	no	3	0	0	III
37	Thyroid	1.2	75	2-10	no	no	yes	no	yes	no	yes	yes	no	1	1b	0	IVA
38	Thyroid	1.8	90	0	<50	no	no	no	no	no	no	no	no	1	0	0	I
39	Thyroid	2.3	90	0	no	NA	yes	yes	yes	no	yes	NA	NA	3	1a	NA	III
40	Thyroid	2.1	90	<2	no	yes	no	no	no	no	yes	yes	no	2	1a	NA	III

ETE: Extra Thyroid Extension; pTNM according the 7th editions UICC; NA: Not Available; NS: Not Search

**Table 3:** Altered miRNA expression in MTC (training set)

miRNAs (miRBase v.12)	miRNAs (miRBase v.20)	log2 Average Exp	Log2 Mean Ratio (T/N)	Adj. *P* value
hsa-miR-375	hsa-miR-375	9.89	6.14	2.64E-27
hsa-miR-136	hsa-miR-136-5p	7.8	3.77	1.49E-17
hsa-miR-487b	hsa-miR-487b-3p	6.15	3.01	3.90E-17
hsa-miR-130a	hsa-miR-130a-3p	9.22	−2.3	4.00E-16
hsa-miR-376c	hsa-miR-376c-3p	7.24	3.67	1.01E-15
hsa-miR-127-3p	hsa-miR-127-3p	6.4	3.6	2.90E-15
hsa-miR-129-3p	hsa-miR-129-2-3p	7.54	4.22	3.69E-15
hsa-miR-199b-5p	hsa-miR-199b-5p	6.92	−2.92	4.23E-15
hsa-miR-30a*	hsa-miR-30a-3p	6.72	−2.45	8.70E-15
hsa-miR-20b	hsa-miR-20b-5p	6.61	−2.18	5.36E-14
hsa-miR-193a-3p	hsa-miR-193a-3p	7.5	−1.6	1.34E-13
hsa-miR-451	hsa-miR-451a	13.22	−3.52	1.57E-13
hsa-miR-200a	hsa-miR-200a-3p	8.93	1.7	2.08E-08
hsa-miR-30a	hsa-miR-30a-5p	9.96	−1.93	1.44E-10
hsa-let-7i	hsa-let-7i-5p	10.69	−2	7.38E-13
hsa-miR-376a	hsa-miR-376a-3p	6.6	2.93	9.17E-13
hsa-miR-10a	hsa-miR-10a-5p	8.26	2.17	1.41E-10
hsa-miR-30c	hsa-miR-30c-5p	9.23	−1.75	2.62E-08
hsa-miR-221	hsa-miR-221-3p	7.26	2.23	6.80E-09
hsa-miR-429	hsa-miR-429	7.83	2.37	7.78E-12
hsa-miR-20a	hsa-miR-20a-5p	8.16	−2.01	3.03E-11
hsa-miR-200b	hsa-miR-200b-3p	9.37	1.45	1.15E-06
hsa-miR-17	hsa-miR-17-5p	7.41	−1.44	1.29E-10
hsa-miR-222	hsa-miR-222-3p	7.67	2.23	5.26E-10
hsa-miR-30e*	hsa-miR-30e-3p	6.38	−1.85	2.53E-10
hsa-miR-144	hsa-miR-144-3p	10.19	−2.68	2.96E-10
hsa-miR-126	hsa-miR-126-3p	10.27	−1.97	6.44E-10
hsa-miR-150	hsa-miR-150-5p	7.33	−2.91	1.04E-09
hsa-miR-223	hsa-miR-223-3p	7.85	−2.2	1.19E-09
hsa-miR-100	hsa-miR-100-5p	9.03	−1.79	1.67E-09
hsa-miR-365	hsa-miR-365a-3p	7.72	−1.42	1.03E-08
hsa-miR-7	hsa-miR-7-5p	10.31	2.74	1.38E-08
hsa-miR-19a	hsa-miR-19a-3p	8.08	−1.35	3.26E-08
hsa-miR-135b	hsa-miR-135b-5p	7.39	−2.01	3.27E-08
hsa-miR-218	hsa-miR-218-5p	6.62	−1.75	3.98E-08
hsa-let-7g	hsa-let-7g-5p	10.31	−1.66	1.91E-07
hsa-miR-335	hsa-miR-335-5p	6.73	2.33	3.04E-07
hsa-let-7f	hsa-let-7f-5p	11.83	−1.43	9.04E-07
hsa-miR-199a-3p	hsa-miR-199a-3p	9.36	−1.95	2.93E-07
hsa-miR-338-3p	hsa-miR-338-3p	7.63	1.02	7.21E-04
hsa-miR-96	hsa-miR-96-5p	6.63	1.66	1.43E-05
hsa-miR-126*	hsa-miR-126-5p	6.51	−1.83	1.49E-06
hsa-miR-214	hsa-miR-214-3p	7.45	−1.51	1.95E-06
hsa-miR-324-5p	hsa-miR-324-5p	6.79	1.1	6.04E-05
hsa-let-7d	hsa-let-7d-5p	8.84	−1.44	5.99E-06
hsa-miR-15b	hsa-miR-15b-5p	9.21	−1.23	6.13E-06
hsa-miR-195	hsa-miR-195-5p	8.62	−1.34	6.80E-06
hsa-miR-181c	hsa-miR-181c-5p	6.35	1.22	6.99E-06
hsa-miR-148a	hsa-miR-148a-3p	8.9	−1.58	7.30E-06
hsa-miR-135a	hsa-miR-135a-5p	9.12	−1.67	4.03E-05
hsa-miR-185	hsa-miR-185-5p	7.17	−1.01	2.74E-05
hsa-miR-199a-5p	hsa-miR-199a-5p	8.3	−1.29	3.04E-05
hsa-miR-301a	hsa-miR-301a-3p	6.93	1.17	1.31E-04
hsa-miR-26b	hsa-miR-26b-5p	10.14	−1.31	1.29E-04
hsa-miR-486-5p	hsa-miR-486-5p	7.78	−1.41	6.46E-05
hsa-miR-557	hsa-miR-557	6.29	1.36	6.46E-05
hsa-miR-142-3p	hsa-miR-142-3p	9.69	−2	7.68E-05
hsa-miR-21	hsa-miR-21-5p	13.06	0.99	2.21E-02
hsa-let-7a	hsa-let-7a-5p	12.17	−1.19	5.32E-04
hsa-miR-146a	hsa-miR-146a-5p	6.77	−1.42	1.02E-04
hsa-miR-143	hsa-miR-143-3p	7.73	−0.99	1.87E-04
hsa-miR-424	hsa-miR-424-5p	8.78	−1.33	1.37E-03
hsa-miR-142-5p	hsa-miR-142-5p	7.13	−1.9	2.49E-04
hsa-miR-10b	hsa-miR-10b-5p	7.09	−1	4.13E-04

### Identification of miR-375-target genes

**Figure 2:**
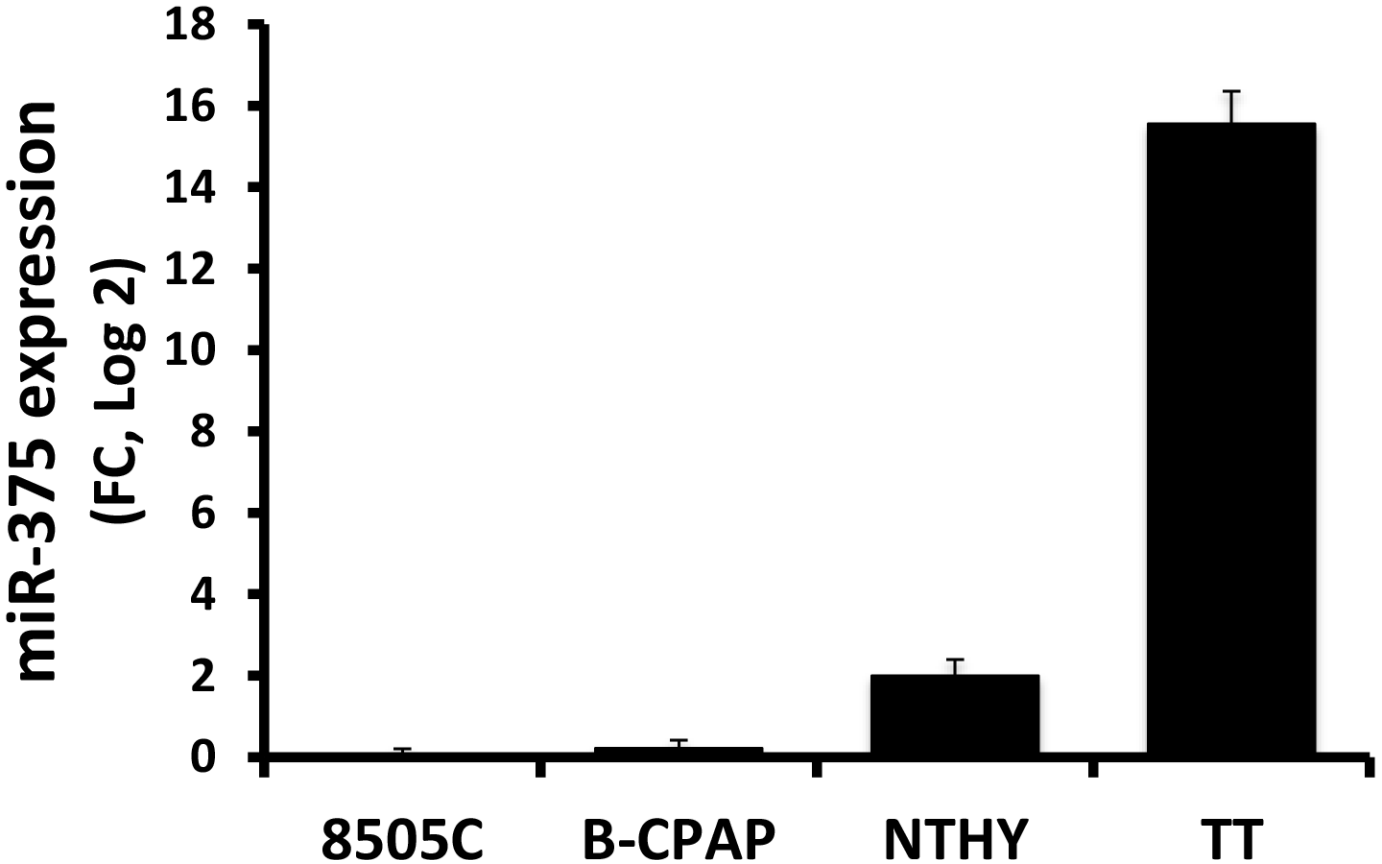
Semi quantitative real-time PCR of miR-375 in thyroid cell lines MiRNA were extracted from 70 percent confluent cells. RNU19 was used for normalization and the 8505C cell line, expressing lowest levels, was used as a reference.

**Figure 3:**
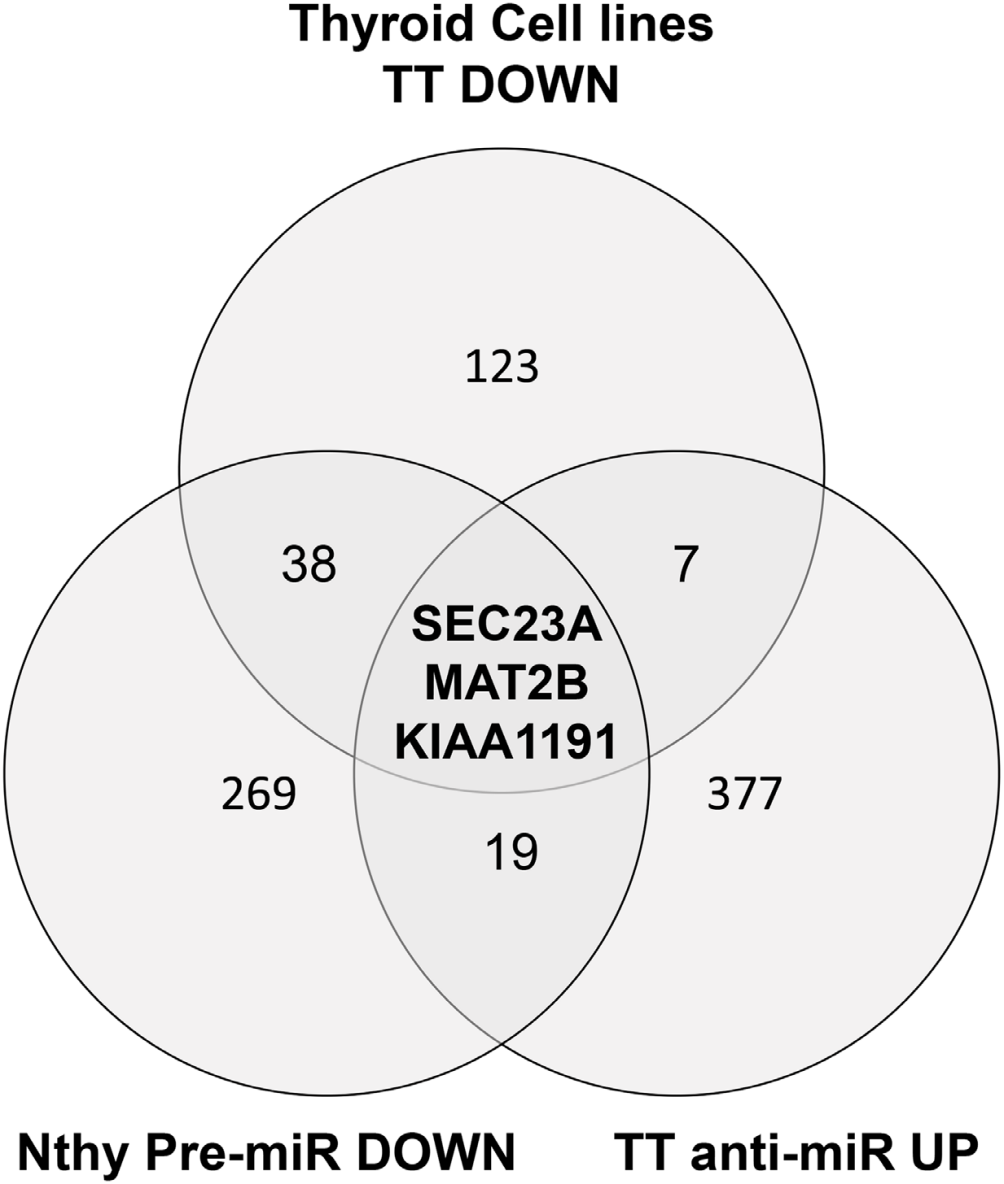
A Venn diagram of the genes passing the cut off filters of 3 independent approaches Nthy-ori 3-1 premiR DOWN: genes that are under-expressed in the Nthy-ori 3-1 after transfection of premir-375. TT anti-miR UP: genes over-expressed in the TT cells transfected with antagomir-375. Thyroid Cell Lines TT_DOWN: genes specifically under-expressed in the TT cell line compared to 11 thyroid cell lines from the public dataset GSE36133. The different cut off values are given in the Material and methods section.

We further focused on miR-375 since it was, by far, the most differentially-regulated miRNA in MTC. We screened miR-375 expression in B-CPAP (papillary thyroid carcinoma cell line), Nthy-ori 3-1 (normal follicular immortalized thyroid cell line), 8505C (thyroid anaplastic carcinoma cell line) and TT thyroid cell lines (HMTC, RET MEN2A) and showed that miR-375 expression was indeed restricted to the TT cell line (Figure 2). We performed miR-375 specific target gene profiling by analyzing the impact of transfection of either a miR-375 mimic or an antagomiR-375 on the transcriptome of Nthy-ori 3-1 control cells or TT cells, respectively. We selected 22 predicted miR-375 target genes (Figure 3) that followed the expected profile in the context of MTC: down-regulated in premiRNA-375 transfected Nthy-ori 3-1 cells and up-regulated in the antagomiR-375 transfected TT cells (Suppl. Table 2).

In parallel, we defined the MTC-specific mRNA expression levels in 12 (5 anaplastic, 5 follicular, 1 papillary, 1 medullary) thyroid cancer cell lines from the CCLE public (dataset GSE36133). Since a high expression level of miR-375 is only found in TT cells, we then selected a set of predicted miR-375 targets (TargetScan V6.0) [32] specifically under-expressed in the TT cell line (Suppl. Table 3: 171 genes). Finally, we retained candidate genes that followed 3 criteria *i*) down-regulated in TT cells *vs* other cell lines and *in silico* predicted to be targeted by miR-375, *ii)* down-regulated in pre-miR-375 transfected Nthy-ori 3-1 cells, and *iii)* up-regulated in the antagomiR-375 transfected TT cells. Overall, our strategy, combining *in silico* and *in vitro* datasets, defined a set of 3 highly relevant candidate genes in the context of MTC: *SEC23A, MAT2B, KIAA1191* (Figure 3).

### Down-regulation of SEC23A is a reliable marker of high miR-375 expression in MTC

**Figure 4:**
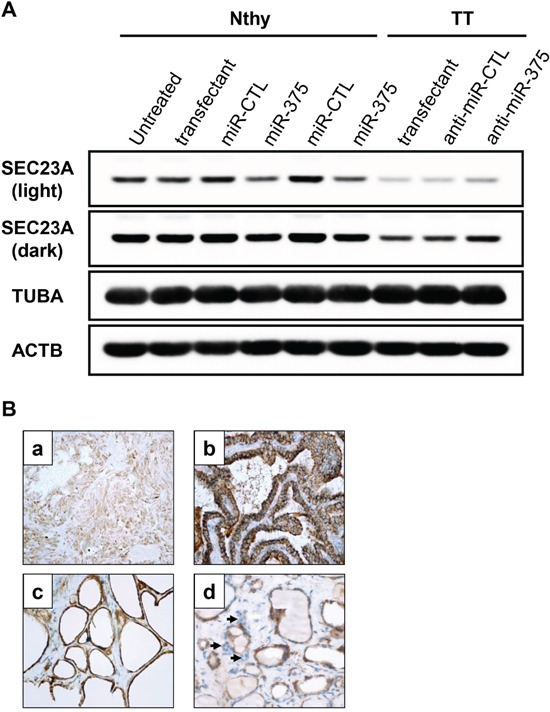
SEC23A expression is negatively associated with miR-375 levels in the thyroid **A.** Nthy-ori 3-1 were transfected with pre-miR-375 or pre-miR-CTL for 48h. TT cells were transfected with antagomiR-375 (anti-miR-375) or antagomiR-CTL (anti-miR-CTL) for 48h. SEC23A protein levels were quantified by immunoblotting. Tubulin (TUBA) and actin B (ACTB) protein levels were used as loading controls. Light exposure (upper band): unsaturated signal for all samples. Dark exposure (lower band): unsaturated signals for TT cells only. **B.** Immunohistochemistry with anti-SEC23A. (a) MTC: weak expression in tumor cells; (b) Papillary thyroid carcinoma: intense cytoplasmic expression in tumor cells; (c) Normal thyroid tissue: intense cytoplasmic expression in normal follicular cells; (d) C-cells: weak expression (arrows). (a-d: immunoperoxdiase, original magnification × 400).

Because SEC23A has been validated as a miR-375 target in a human prostatic carcinoma cell line [33], we further validated SEC23A expression at the protein level in MTC by immunoblotting and immunohistochemistry. The SEC23A protein expression level was low in TT cells in comparison with Nthy-ori 3-1 cells (Figure 4). As expected, the SEC23A levels decreased after transfection with a miR-375 mimic (Figure 4A), while SEC23A expression was increased in TT cells transfected with the antagomiR-375, according to the microarray results. Immunohistochemical analysis of thyroid tissues confirmed the decreased in SEC23A cytoplasmic expression in MTC sections and C-cells when compared to non-tumor tissues and non-MTC thyroid carcinomas (Figure 4B). In conclusion, these results suggested an inverse correlation between miR-375 and SEC23A expression *in vitro* and *in vivo*.

### MiR-375 is associated with a decreased cell proliferation and improved sensitivity to vandetanib treatment

**Figure 5:**
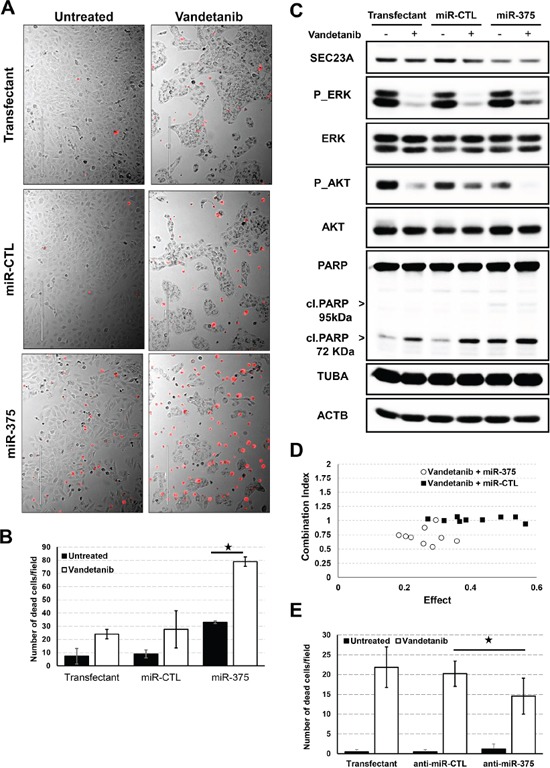
Effect of miR-375 on proliferation and cancer drug response **A.** Nthy-ori 3-1 cells were seeded and transfected with pre-miR-375 or pre-miR-CTL at 20pM for 24h and vandetanib was then added for 48h. Dead cells were stained with propidium iodide (red) before microscopic analysis. Pictures representative of four biological replicates. **B.** Quantification of propidium iodide positive Nthy-ori 3-1 cells. **C.** Nthy-ori 3-1 cells were seeded and transfected with pre-miR-375 or pre-miR-CTL at 20pM for 24h and vandetanib was then added for 48h. Quantification of ERK, AKT pathways and PARP cleavage was performed by immunoblotting. Tubulin (TUBA) and actin B (ACTB) protein levels were used as loading controls. **D.** Nthy-ori 3-1 cells were seeded in 96-well plates and transfected with pre-miR-375 or pre-miR-CTL either at 6.25, 12.5, 25 pM for 24h and then treated with either 1.25, 2.5, 5μM vandetanib for 48h. Cell proliferation was evaluated using BrdU incorporation for 3h. Single doses and combination doses were analysed using Compusyn software and a Combination index/effect dot plot was generated. CI<1 values are indicative of synergism. **E.** TT cells were seeded and transfected with antagomiR-375 (anti-miR-375) or antagomiR-CTL (anti-miR-CTL) for 24h and vandetanib was then added for 48h. Quantification of propidium iodide positive TT cells.

We then investigated the effect of miR-375 on the cell cycle. For this, we generated a cell cycle-reporter (Nthy-ori 3-1 FUCCI-2A) cell line and transfected miR-375. Expression of miR-375 in Nthy-ori 3-1 cells decreased cell proliferation after 24h as shown by both a reduction in S/G2 of the cell cycle together with an increase in the percentage of cells in G1 (Suppl. Figure 1). At 72 h, this anti-proliferative effect was easily visualized by microscopy with a strong difference of confluence between miR-375 transfected cells and control cells (Figure 5A). Moreover, miR-375 transfected cells showed increased mortality as evidenced by a significant increase in the number of dead cells (Figure 5B) and in PARP cleavage (Figure 5C). PARP cleavage resulted in a fragment at 72 kDa, which may be compatible with activation of calpains [34], rather than the expected caspase cleavage at 95 kDa.

Vandetanib is a tyrosine kinase inhibitor currently prescribed for the treatment of advanced MTC [10, 35]. It acts as a kinase inhibitor of a number of cell receptors, mainly the vascular endothelial growth factor receptor (VEGFR), the epidermal growth factor receptor (EGFR), and the RET-tyrosine kinase (wild-type and mutant). The MAPK (Ras/Mek1-2/p44-42), and PI3K/AKT pathways have been described to be the two major signaling pathways inhibited by vandetanib. In our system, vandetanib consistently decreased the phosphorylation of ERK (MAPK1 and 3) and AKT/PKB together with accumulation of the cleaved form of PARP and a decrease in Nthy-ori 3-1 cell proliferation.

Transfection of miR-375 sensitized the cells to the drug, as shown by a stronger decrease in proliferation and pronounced increase in dead cells (Figure 5A, 5B and 5C) (*P*-value 5 × 10^−5^). This effect was also associated with strong inhibition of the phosphorylated form of AKT/PKB and increased accumulation of cleaved PARP.

The cytotoxic combination of miR-375 and vandetanib on proliferation was further evaluated using BrdU cell proliferation assay. The synergistic effect (combination index CI < 1) on proliferation reached a maximum CI of 0.54 at the concentration of 2.5μM of vandetanib and 6.25μM of miR-375. In contrast, the miR-CTL and vandetanib had an additive effect on proliferation (Figure 5D).

Moreover, to demonstrate that endogenous levels of miR-375 were sufficient to mediate this effect, TT cells were transfected with antagomiR-375 and treated with the drug. AntagomiR-375 significantly reduced the mortality of TT cells induced by vandetanib compared to either antagomiR-CTL or lipofectamine alone (Figure 5E). We furthermore confirmed that vandetanib strongly inhibited AKT and ERK signalling. However, as previously described [36], vandetanib had no effect on PARP cleavage in the TT cell line (Suppl. Figure 2).

**Figure 6:**
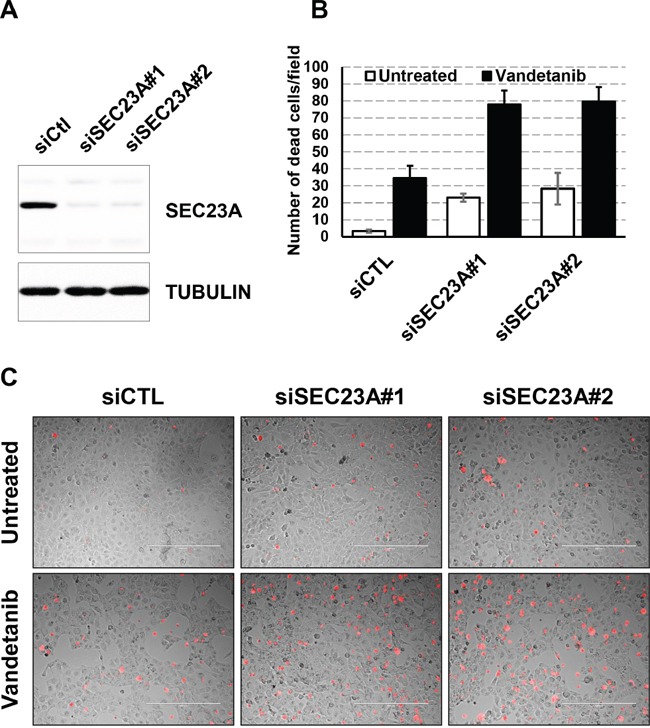
Effect of siSEC23A on the vandetanib response **A.** Nthy-ori 3-1 cells were seeded and transfected with siCTL or siSEC23A#1 or siSEC23#2 for 48h. SEC23A protein levels were quantified by immunoblotting. Tubulin (TUBA) protein levels were used as a loading control. **B.** Nthy-ori 3-1 cells were seeded and transfected with siSEC23A or siCTL for 24h and vandetanib was then added for 48h. Dead cells were stained with propidium iodide (red) before microscopic analysis. Quantification of propidium iodide positive cells. **C.** Pictures representative of four biological replicates.

We finally investigated the role of SEC23A in this mechanism. SEC23A was silenced (siSEC23A) in Nthy-ori 3-1 cells using 2 different siRNAs. Decreased proliferation and increased toxicity was observed in cells silenced for SEC23A, in line with the pro-apoptotic effect of miR-375. Furthermore, we also found increased cell mortality in the presence of vandetanib, underscoring that SEC23A down-regulation is likely associated with the miR-375-mediated sensitization of vandetanib (Figure 6).

Together, these results demonstrated that the miR-375/SEC23A axis acts as a regulator of thyroid tumor cell proliferation and synergistically potentiates the therapeutic effect of vandetanib.

## DISCUSSION

The role of miRNA in thyroid carcinogenesis has been extensively studied in recent years [19]. However, most of the previous datasets describing miRNA expression profiling in MTC are based on a limited number of patients [20–25, 37, 38] (Suppl. Table 5). In the present studied cohort of 62 MTC patients, we showed up-regulation of miR-375, -129, -10a, and down-regulation of miR-451 in tumor *vs* non-tumor tissues, overlapping with results produced by different previously published studies [20–22, 37]. We also found a slight up-regulation of miR-21 in tumor tissue, in agreement with a recent study of 64 MTC patients [26]. However, our overlap with other studies is partial and might be explained by technical bias including the miRNA quantification methods or differences in the design of the experiment. However, our approach, based on paired analysis of tumor *vs* non-tumor samples from the same patient may offer a more robust methodology for the control of inter-individual variation [20–22, 37].

MiR-375 was the most significant up-regulated miRNA in our cohort and its gradual increase in expression from non-tumor-adjacent tissue to hyperplasia to MTC was consistent with previous observations [21]. Using an approach combining transcriptome analyses of miR-375 activation and inhibition as well as data exploration of the Cancer Cell Line Encyclopedia (CCLE) transcriptome database, we established SEC23A as a reliable miR-375 target, in accordance with previous reports in prostate carcinomas [33, 39]. Using immunoblotting and immunohistochemistry, we confirmed the under-expression of SEC23A in the presence of miR-375 in TT cells and in MTC compared to non-tumor adjacent thyroid tissue.

Previously, miR-375 was found to be significantly down-regulated in various types of cancers, suppressing core hallmarks of cancer by targeting several important oncogenes, such as PDK1, JAK2, AEG-1 [40]. However, over-expression of miR-375 has also been reported in breast and prostate cancer [33, 39, 41–43]. In breast cancers, it has been reported that down-regulation of miR-375 by antisense RNA inhibited the proliferation of the breast cancer cell line MCF-7 without inducing apoptosis [41], while increased expression of hsa-miR-375 in normal breast epithelium resulted in a loss of cellular organization and acquisition of a hyperplastic phenotype [42]. In *in vitro* experiments, miR-375 over-expression resulted in a decrease in proliferation and an increase in apoptosis of the transfected cells compared to control cells. This effect was mediated through SEC23A since siSEC23A were also associated with decreased proliferation and increased cell death. The role of miR-375 in cancer is still unclear but the level is increased in several pathologies including MTC due to its anti-proliferative and sometimes pro-apoptotic action. These opposing effects could be due to differences in the type of mRNA targeted by miR-375 of different tissues. It may also be possible to hypothesize that miR-375 may act as passenger miRNA expressed at the same time as strong oncogenes that counteract the action of miR-375.

Vandetanib (ZD6474, trade name CAPRELSA) is a tyrosine kinase inhibitor [44]. The MAPK (Ras/Mek1-2/p44-42), and PI3K/AKT pathways have been described to be the two major signaling pathways inhibited by vandetanib [45]. Both the FDA (Food and Drug Administration) and EMEA (European Medicines Agency) have approved vandetanib for treatment of patients with recurrent or metastatic MTC that are unresectable, and/or symptomatic [9]. However, a better understanding of the pathways involved in this treatment may help prevent disease relapse and/or treatment resistance. Interestingly, miR-375 over-expression synergistically increased the sensitivity of transfected cells to vandetanib, with a stronger decrease in cell proliferation associated with a large increase in dead cells in transfected cells compared to control cells. Interestingly, the decrease in SEC23A was sufficient to obtain vandetanib sensitivity, since siSEC23A also gave a stronger vandetanib response. Since SEC23A is involved in anterograde transport from the ER to the Golgi, the silencing of SEC23A and the subsequent blockade of secretory traffic finally may result in Golgi collapse, calpain dependent activation of caspase and cell death. Taken together, our data indicate that the expression levels of miR-375 or SEC23A may be good predictive indicators for better use of vandetanib in MTC.

In summary, we demonstrated that over-expression of miR-375 in MTC resulted in decreased SEC23A protein expression in tumor tissue. We propose that miR-375 over-expression associated with SEC23A down-regulation could improve the efficacy of vandetanib in targeting of tumor cells, constituting an alternative route for controlling cell proliferation.

## MATERIALS AND METHODS

### Patients and tissue samples

62 MTC from patients with well-documented clinical follow-up were included in this study. A set of 40 MTC was used for microarray analysis (training set) and a set of 22 MTC was used for validation of miRNAs of interest (validation set). Tissue specimens were collected from five related hospital biobanks in France (Bordeaux, Marseille, Nice, Paris, Reims). Tissue specimens (from both tumor and non-tumor thyroid tissue) were immediately frozen in nitrogen after surgical resection and stored at −80°C until use. Mirror tissue samples of the frozen specimens were fixed in formaldehyde and stained with hematoxylin eosin for histological assessment of the percentage of tumor cells and the absence of associated C cell hyperplasia in the non-tumor adjacent tissue samples. All patients provided a signed informed consent for participation in the study and the protocol was approved by the local ethics committee of each university participating in the study.

### MiRNA microarrays

Total RNA was extracted from samples with TRIzol solution (Invitrogen, Carlsbad, CA, USA), and the integrity of the RNA was assessed using an Agilent Bioanalyzer 2100 (Agilent Palo Alto, CA). Total RNA (100 ng) was labeled and hybridized onto Agilent Human miRNA Microarrays V2 (miRBase release 10.1, Platform GPL8227 in GEO: http://www.ncbi.nlm.nih.gov/geo) or V3 (miRBase release 12.0, Platform GPL10850 in GE0) according to the manufacturer's protocol, and scanned using the Agilent Microarray Scanner. The scanned images were processed by Agilent's Feature Extraction software version 9.5.3. All probes were associated with the most recent miRBase release annotations (v12.0). Normalization was performed using the Limma package available from Bioconductor [27]. Inter slide normalization was performed using the quantile method followed by log2 transformation, after addition of a small constant (10), such that the smallest value of the data set was 10.1 before taking the log. Means of ratios from all comparisons were calculated and a Student's *t*-test analysis was performed. The Benjamini-Hochberg procedure was used to control the experiment-wise false discovery rate (FDR) from multiple testing procedures. Genes with a log2 average expression value superior or equal to 6, an absolute log2 fold-change superior to 1.0, and an adjusted p-value inferior to 0.05 are considered differentially expressed. Hierarchical clustering was performed on the logFC expression values of the 64 differentially expressed miRs using GenePattern [28]. The eucledian distance measure and the complete clustering method were used both on the patients and the genes. The experimental data have been deposited in the NCBI Gene Expression Omnibus (GEO) (http://www.ncbi.nlm.nih.gov/geo/) under Serial record GSE40807.

### MiRNA quantitative real-time RT-PCR analysis

For the validation set: the expression of the miRNA candidates identified in the training set was further evaluated with a second independent set of 22 MTC samples. Quantitative real-time RT-PCR was performed for the validation set to check for the expression of the 2 selected miRNAs of interest (miR-375 and miR-451), according to the manufacturer's protocol (Applied Biosystems, SD, CA). The relative expression level was calculated for each sample after normalization against the endogenous expression level of the housekeeping miRNA RNU-19 and the corresponding non-tumor tissue, using the ΔΔCt method for comparison of relative fold-expression differences. Statistical analysis was performed using the unpaired two-tailed Student's *t*-test. Statistical significance was defined as a *P*-value of <0.05. Kits for amplification are listed in the supplementary data (suppl. Table 1).

For FFPE MTC and C cell hyperplasia: we selected 6 patients having a MTC with associated C cell hyperplasia. We defined for each patient, on HE slides, for macrodissection, a MTC area, a C cell hyperplasia area, and a normal tissue area. For each selected area, the percent of C-cell was measured thanks to calcitonin and haematoxylin staining. The mean quantification of two pathologists (SL, MI) is presented in supplementary Table 4.

MiRNA extraction from FFPE material was done using a miRNeasy FFPE kit, according to the manufacturer's protocol (Qiagen). Quantitative real-time RT-PCR was then performed for the expression of the miR-375 as described earlier using normalization to the housekeeping miRNA RNU-19. This expression value was then adjusted to the percentage of C-cell positive staining. Finally, each log2(expression value) was corrected using the median of non-tumor tissue (ΔΔCt). The CT value and % of C-positive staining are presented in supplementary Table 4.

### Cell culture

Nthy-ori 3-1 (non-tumor follicular cell line, ECACC, catalogue number 90011609), TT cells (medullary carcinoma, ATCC® CRL-1803™), 8505C (Anaplasic carcinoma, DSMZ, ACC-219), B-CPAP (Papillary carcinoma, DSMZ, ACC-273) were grown in appropriate media supplemented with 10% fetal calf serum, sodium pyruvate and penicillin/streptomycin (Life Technologies) for less than 25 passages. All cell lines were authenticated by determining the genetic characteristics by PCR-single-locus-technology (Promega, PowerPlex 21 PCR Kit) and certified (Eurofins, Eurofins Genomics, Ebersberg, Germany). Vandetanib (Caprelsa, AstraZeneca, London) was residual material given to patients of the Centre Antoine Lacassagne. Vandetanib was dissolved in DMSO and used at 10μM for 48h. Treated cells showed induced cytotoxicity as determined by contrast phase microscopy and propidium iodide staining (5μg/ml) of late apoptotic cells. Dead cells were counted using ImageJ software (NIH) (particle analysis, size 100-1000, circularity 0.3-1).

### siRNA, miRNA mimics and antagomiR transfection

TT cells were transfected with antagomiR-375 (ref. MH10327) or antagomiR-CTL (ref. 4464076), and Nthy-ori 3-1 cells with pre-miR-375 (ref. MC10327) or pre-miR-CTL (ref. 4464058) or siSEC23A (ref. ID135698, 135699) or siCTL (ref. AM4611) (all purchased from Life Technologies, France) as previously described [15]. Cells were plated at 100 000 cells/well in a six-well plate and transfected for 48h with synthetic pre-miRs, antagomiRs or siRNA at a final concentration of 50, 200, or 50 nM respectively using Lipofectamine RNAiMAX reagent (Life Technologies), following the manufacturer's instructions. The level of transfection was checked by RT-PCR for the specific transfected miRNA on AB7500 thermal cycler (Applied Biosystems).

### Transcriptome microarray analysis

Total RNA of TT or Nthy-ori 3-1 cells transfected for 48h with either pre-miR-CTL, pre-miR-375 or antago-miR-375 was extracted using the RNeasy kit (Qiagen, Hilden, Germany). The integrity of the RNA was assessed using an Agilent BioAnalyzer 2100 (Agilent Technologies). RNA samples were then labeled and hybridized on 8×60K high density SurePrint G3 gene expression human Agilent microarrays following the manufacturer's instructions. Two or three biological replicates were performed for each experimental condition. The microarray experimental data were deposited in the NCBI GEO under the serial record number GSE67742.

The data were quantile normalized using the Bioconductor package limma [27]. Means of ratios from all comparisons were calculated and the moderated t-statistic of the limma package provided the per gene *P*-values. The Benjamini-Hochberg procedure was used to control the experiment-wise false discovery rate (FDR) from multiple testing procedures. Differentially expressed genes were analyzed based on two contrasts, pre-miR-375 *versus* the pre-miR-CTL transfection in the Nthy-ori 3-1 cells and the antagomiR-375 transfection *versus* the CTL in the TT cells. Down-regulated genes in Nthy-ori 3-1 pre-mir-375 *versus* control were selected based on a log2 average expression value superior or equal to 6, a log2 fold change value inferior or equal to −1, and an adjusted P-value inferior or equal to 0.05. The selection of genes up-regulated in TT antagomir-375 versus control was based on a log2 average expression value superior to 6 and a log2 fold change superior or equal to 0.6.

We downloaded the normalized gene expression values of 12 thyroid cancer cell lines extracted from the publicly available GEO (accession number GSE36133) [29]. Among all the genes under-expressed in the TT cell line compared to all the other 11 thyroid cancer cell lines (follicular origin), we selected those with an average log2 expression level superior or equal to 8 and a log2 fold change inferior or equal to −1.

### Immunoblot analysis

Protein extracts were run as previously described [15]. Membranes were incubated either with rabbit anti-SEC23 antibody (1:5,000, AB137583, Abcam, USA), rabbit anti-PARP antibody (E78) (1:1,000, AB32071, Abcam), rabbit anti-phospho-AKT (Ser473) (1:1,000, 9271S, Cell Signaling Technology (CST), USA), rabbit anti-AKT (1:1,000, 9272S, CST), rabbit anti-phospho-p44/42 MAPK (ERK1/2) (Thr202/Tyr204) (1:1,000, 9101S, CST), rabbit anti-p44/42 MAPK (ERK1/2) (1:1,000, 9102S, CST), mouse anti-tubulin (1:10,000, DM1A clone, T9026, Sigma Aldrich, USA) and mouse anti-actin (1:10,000, AC-40 clone, A3853, Sigma Aldrich) antibodies as loading controls. Antibodies were detected with a HRP-conjugated anti-mouse or anti-rabbit antibody (1:6,000, Santa Cruz Biotechnology, USA) using the Enhanced Chemiluminescence detection system (Pxi, Syngene). Signals presented thoroughout the study were captured before saturation of all samples (light exposure), however, for Figure 4, we choose to present a stronger exposure in order to better observe differences in TT cell lines (unsaturated signals).

### SEC23A immunohistochemistry

Anti-SEC23A immunostaining was performed on 3μm-thick whole tissue sections of 11 MTC FFPE tumors and 13 thyroid follicular tumors (8 papillary carcinomas and 4 follicular carcinomas), using the polyclonal rabbit antibody provided by Abcam (AB137583). Immunostaining was performed with a Ventana® Benchmark immunostainer (Roche Diagnostics, Meylan, France) using the Ventana Ultraview detection kit, following the manufacturer's procedure (CC2© pre-treatment for 60min, antibody dilution at 1:200, incubation at 37°C for 32min, UltraView detection kit© without UltraView Amplification©). Slides were freshly cut less than two weeks before IHC technique and stored at 4°C before use. SEC23A antibody staining was blindly analyzed by three pathologists (S.L, V.H. and E.L).

### Nthy-ori 3-1 FUCCI-2A cell line and cell cycle analysis

Replication-defective, self-inactivating retroviral constructs were used to establish a stable Nthy-ori 3-1 FUCCI-2A cell line. The pPRIPu CrUCCI plasmid was obtained from Céline Feillet and Frank Delaunay and cell lines were generated as previously described [30]. Based on their fluorescence, single cells were analyzed post-transfection using a BD FACS ARIA (Becton Dickinson). Non-marked: Early G1, Kusabira-Orange 2 (mKO2) only: G1, mKO2 + Azami-Green 1 (mAG1): Early S, mAG1 only: S/G2/M. mKO2 and mAG1 were excited with 561 nm and 488 nm laser lines, respectively. Fluorescence was collected at 585 nm (585/15 BP) for mKO2 and at 530 nm (530/30 BP) for Geminin. The experiment was done in triplicate.

### Proliferation assay using BrdU incorporation

A chemiluminescent immunoassay for the quantification of cell proliferation was performed based on the measurement of BrdU (Roche Diagnostics, Penzberg, Germany). At Day 0, 2.000 cells were plated in a 96-well plate. After miRNA and/or vandetanib treatment, cells were incubated with BrdU (10 μM) for 3h, then fixed and denatured. Cells were subsequently treated with a peroxidase-labeled anti-BrdU antibody for 90min and the chemiluminescent reaction was performed. All assays were performed in quadruplicate and were repeated twice under independent conditions. Data are presented as means ± SEM.

### Statistical analysis

The Prism6 program was used for statistical analysis (Graphpad Software, La Jolla, CA, USA). The results were evaluated for statistical significance by the Student's t-test or the ANOVA test. Error bars represent the S.D. of the mean. *P*-values <0.05 were regarded as significant.

Drug synergy was measured using the CompuSyn software (www.combosyn.com/). To perform these analyses, we used the data obtained from the growth inhibitory experiments, which generated isobolograms and measured the combination index (CI) based on the median-effect principle of Zhang et al. [31]. The CI method is a mathematical representation that measures a two-drug pharmacological interaction. A CI of 1 indicates an additive effect between two agents, a CI <1 indicates synergism, whereas a CI >1 represents antagonism.

## SUPPLEMENTARY FIGURES AND TABLES






