# Supplementary material for: MicroRNA-375/SEC23A as biomarkers of the in vitro efficacy of vandetanib
Source: Oncotarget. 2016 Mar 29;7(21):30461–78. doi: 10.18632/oncotarget.8458 (PMC5058693; doi:10.18632/oncotarget.8458)
Supplement: Supplementary file 1 [file oncotarget-07-30461-s001.pdf]

# MicroRNA-375/SEC23A as biomarkers of the *in vitro* efficacy of vandetanib

## SUPPLEMENTARY FIGURES AND TABLES

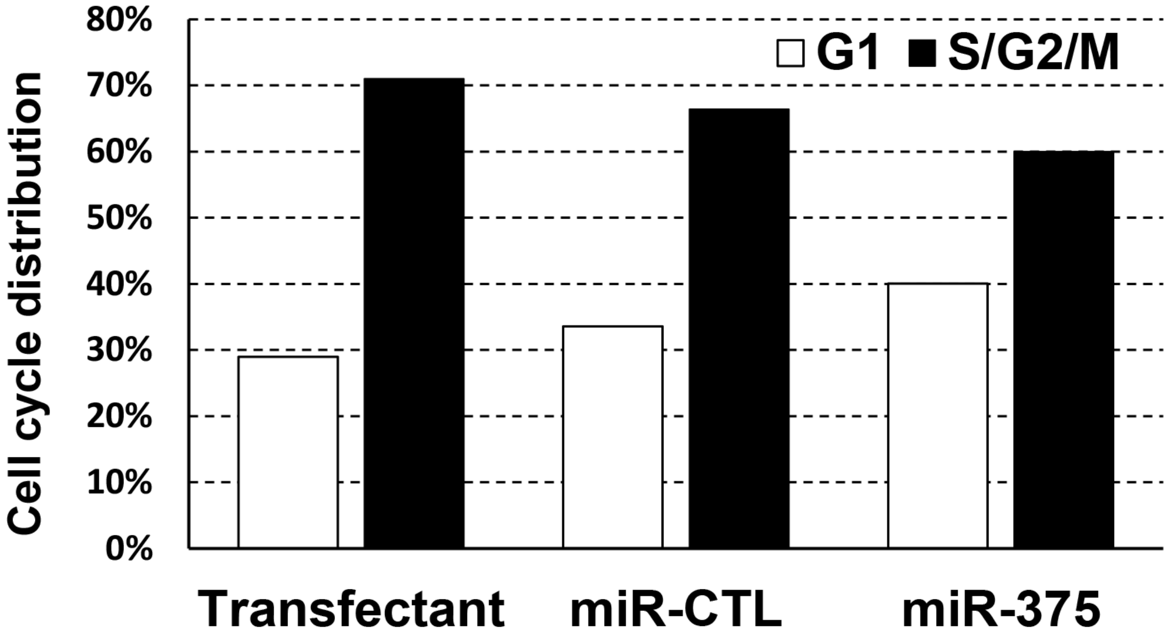


**Supplementary Figure S1: Nthy-ori 3-1 - FUCCI2A cells were seeded and transfected with pre-miR-375 or pre-miR-CTL at 20pM for 24h.** Cells were then harvested and analyzed by flow cytometry as described in the Material and methods.

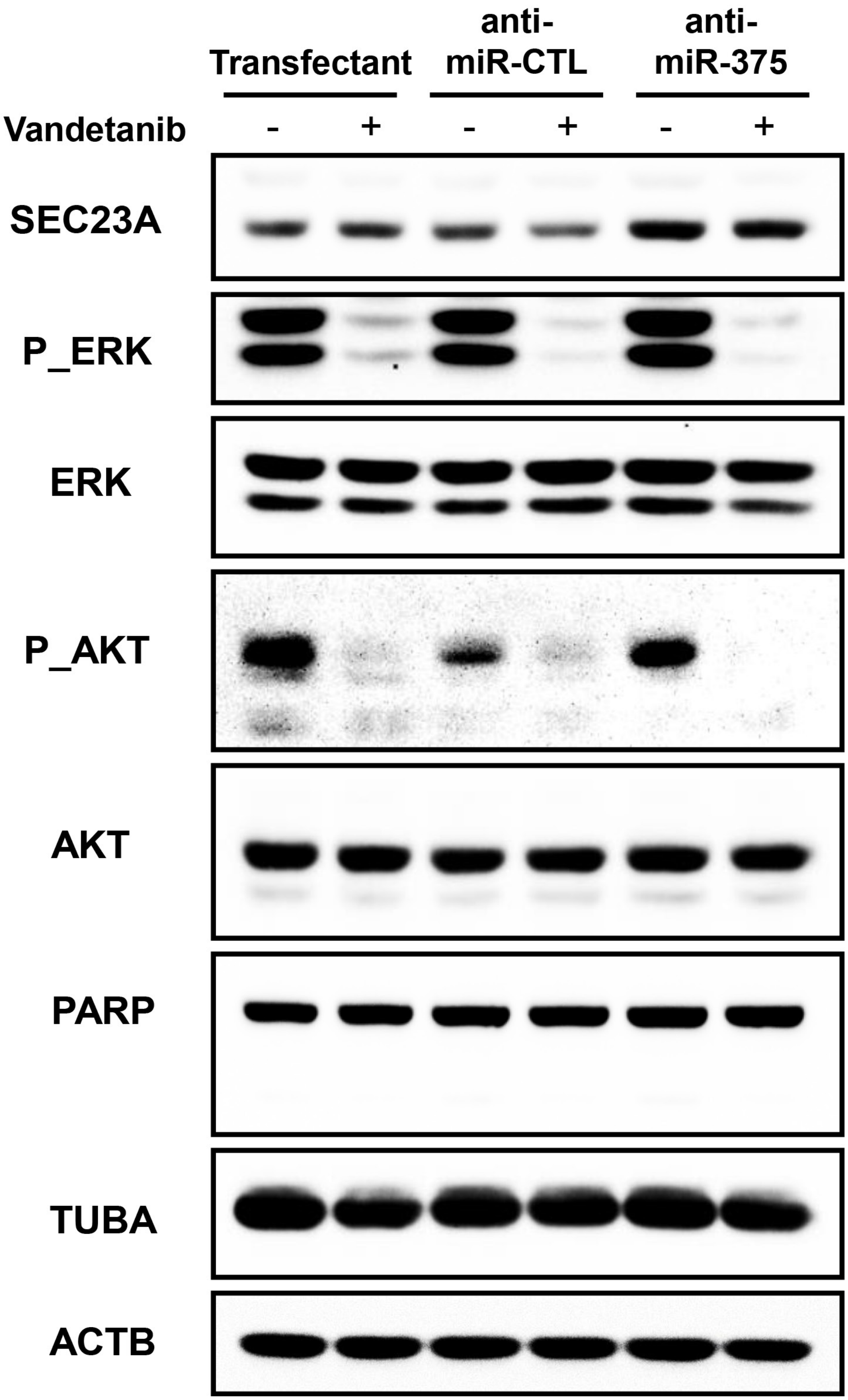


**Supplementary Figure S2: TT cells were seeded and transfected with antagomiR-375 (anti-miR-375) or antagomiR-CTL (anti-miR-CTL) for 24h and vandetanib was then added for 48h.** Quantification of propidium iodide positive TT cells. Quantification of ERK, AKT pathways and PARP cleavage was performed by immunoblotting. Tubulin (TUBA) and actin B (ACTB) protein levels were used as loading controls.

**Supplementary Table S1: List of different primers used for qPCR analysis**

| MicroRNAs | Target sequences | Applied references |
|---|---|---|
| Hsa-miR-375 | UUUGUUCGUUCGGCUCGCGUGA | 000564 |
| Hsa-miR-451 | AAACCGUUACCAUUACUGAGUU | 001105 |
| RNU 19 | TTGCACCTCTGAGAGTGGAATGACTCCTGTGGAGTT GATCCTAGTCTGGGTGCAAACAATT | 001003 |

**Supplementary Table S2: The transcriptome microarray expression profile for the set of 22 genes (24 probe-sets) down-regulated in NThy-ori 3-1 pre-mir-375 transfected cells and up-regulated in the TT antagomir-375 transfected cells**

See Supplementary File S1

**Supplementary Table S3: Genes under-expressed in the TT cell line and predicted as strong targets of miR-375**

See Supplementary File S2

**Supplementary Table S4: Overview of the studies that performed a miRNA analysis of MTC**

| Authors | Results | Prognostic value |
|---|---|---|
| Abraham *et al*. 2011 | Over-expression of miR-182, -183, -375, -551b and down-regulation of miR-199b-5p, -9, -9*, -223, let-7i, miR-23a in SMTC vs HMTC | miR-183 and -375 associated with<br>- lymph node metastases<br>- residual disease<br>- mortality |
| Duan *et al*. 2014 | Down-regulation of miR-129-5p | |
| Gundara et al. 2014 | Primary tumor and nodal metastases miRNA relative expression correlation for miR-9*, -183 and -375 | |
| Hudson *et al*. 2013 | Over-expression of miR-375, -10a and down-regulation of miR-455 | |
| Mian *et al*. 2012 | Over-expression of miR-21, -127, -154, -224, -323, -370, -183, -375, -9* | miR-224 associated with<br>- absence of lymph node metastases<br>- lower stages at diagnosis<br>- biochemically-free status at the end of the follow-up |
| Nikiforova *et al*. 2008 | Over-expression of miR-323, -370, -129, -137, -10a, -124a, 224, 127, -9, -154. | |
| Pennelli *et al*. 2015 | Over-expression of miR-21 | Associated with high calcitonin levels, lymph node metastases, advanced stages, biochemically persistent disease |
| Santarpia *et al*. 2013 | Over-expression of miR-130a, -138, -193a-3p, -373, -498 and down-regulation of miR-10a, -200b/-200c, -7, -29c in metastatic MTC | |

**Supplementary Table S5: Overview of miRNA studies performed on MTC**

| | % C-Cells | | | ΔCT (CT-RNU19) | | |
|---|---|---|---|---|---|---|
| **Patients** | **Non tumoral tissue** | **C-Cell Hyperplasia** | **MTC** | **Non tumoral tissue** | **C-Cell Hyperplasia** | **MTC** |
| Patient 1 | 0 | 20 | 90 | 3.68 | 9.36 | 13.40 |
| Patient 2 | 0 | 5 | 40 | 2.40 | 3.28 | 4.35 |
| Patient 3 | 0 | 10 | 90 | 0.25 | 5.31 | 12.49 |
| Patient 4 | 0 | 10 | 70 | 2.97 | 4.17 | 12.66 |
| Patient 5 | 0 | 3 | 90 | 4.03 | 4.25 | 13.09 |
| Patient 6 | 0 | 20 | 70 | 3.70 | 7.92 | 12.02 |
